# Microbiota fingerprints within the oral cavity of cetaceans as indicators for population biomonitoring

**DOI:** 10.1038/s41598-019-50139-7

**Published:** 2019-09-23

**Authors:** Pedro Soares-Castro, Helena Araújo-Rodrigues, Filipa Godoy-Vitorino, Marisa Ferreira, Pablo Covelo, Alfredo López, José Vingada, Catarina Eira, Pedro Miguel Santos

**Affiliations:** 1 Department of Biology and Centre for Molecular and Environmental Biology (CBMA), University of Minho, Campus de Gualtar, 4710-087 Braga, Portugal; 2 University of Puerto Rico, School of Medicine, Department of Microbiology and Medical Zoology, Microbial Ecology and Genomics Lab, GPO Box 365067, San Juan, Puerto Rico 00936-5067 USA; 3 Portuguese Wildlife Society (SPVS), Quiaios, Field Station, Apartado 16 EC Quiaios, 3081-101 Figueira da Foz, Portugal; 4 Coordinadora para o Estudo dos Mamíferos Mariños (CEMMA), P.O. Box 15, 36380 Pontevedra, Gondomar Spain; 5 Department of Biology and CESAM, University of Aveiro, 3810-193 Aveiro, Portugal

**Keywords:** Microbial ecology, Metagenomics, Microbiome, Food webs

## Abstract

The composition of mammalian microbiota has been related with the host health status. In this study, we assessed the oral microbiome of 3 cetacean species most commonly found stranded in Iberian Atlantic waters (*Delphinus delphis*, *Stenella coeruleoalba* and *Phocoena phocoena*), using 16S rDNA-amplicon metabarcoding. All oral microbiomes were dominated by *Proteobacteria*, *Firmicutes*, *Bacteroidetes* and *Fusobacteria* bacteria, which were also predominant in the oral cavity of *Tursiops truncatus*. A Constrained Canonical Analysis (CCA) showed that the major factors shaping the composition of 38 oral microbiomes (p-value < 0.05) were: (i) animal species and (ii) age class, segregating adults and juveniles. The correlation analysis also grouped the microbiomes by animal stranding location and health status. Similar discriminatory patterns were detected using the data from a previous study on *Tursiops truncatus*, indicating that this correlation approach may facilitate data comparisons between different studies on several cetacean species. This study identified a total of 15 bacterial genera and 27 OTUs discriminating between the observed CCA groups, which can be further explored as microbiota fingerprints to develop (i) specific diagnostic assays for cetacean population conservation and (ii) bio-monitoring approaches to assess the health of marine ecosystems from the Iberian Atlantic basin, using cetaceans as bioindicators.

## Introduction

The ecological sustainability of aquatic environments is being dramatically threatened by climate change, habitat deterioration and a vast array of human-driven activities. Human impacts affect directly ocean life, often leading marine species to an endangered status^1–3^. Cetaceans, particularly the Odontoceti, occupy high trophic levels (predators, primary or secondary consumers) and, consequently, their condition and wellbeing reflect the health and status of lower trophic levels^4–7^.

The common dolphin (*Delphinus delphis*) is one of the most abundant cetacean species in Atlantic Iberian waters^8^, sharing the habitat with other members of the family Delphinidae, such as the bottlenose dolphin (*Tursiops truncatus*) and the striped dolphin (*Stenella coeruleoalba*). The more coastal delphinids also share their habitat with the harbour porpoise (*Phocoena phocoena*), a member of the family Phocoenidae. The highest abundances of striped dolphins in Portugal are markedly found offshore, whereas harbor porpoises are mostly concentrated near the coast. On the other hand, important common dolphin abundances can be found both near the coast and offshore, sometimes forming mixed groups with striped dolphins^9^.

Recent surveys showed that the harbour porpoise population is severely declining in Iberian Atlantic waters^9^. There is a growing concern about the impact of various hazards to marine wildlife, and recent EU legal frameworks (2008/56/EC, 2010/477/EU, 2017/848/EU) highlight the need for marine populations to achieve Good Environmental Status. The more near-coastal animals may be exposed to a wide variety of anthropogenic contaminants and pathogens. Systematic studies on marine mammal health and disease are crucial to support conservation and management measures, particularly considering species such as the harbour porpoise.

In Continental Portugal, since the year 2000 when regional dedicated marine animals stranding networks started operating until 2016, a relatively high cetacean stranding rate was registered. The annual average rates amount to 236 stranded cetaceans, even though only about 500 km of the Portuguese coast has been monitored on a regular basis and the major cause for dead strandings (nearly 45%) was accidental capture^9^ and also confirmed disease (e.g. viral and bacterial infections^10–12^). However, in many cases (about 36%), the observed cetacean mortality causes remained undetermined, due to the number of stranded animals found in advanced decomposition stages.

Mammalian microbial communities have critical roles in the nutrition of the host, resistance to colonization by pathogens and the maturation of the host immune system^13,14^. Moreover, the microbiota composition of each animal species is shaped by the interactions with the immune system, according to the animal physiology, diet and social habits, environmental context, among others^15^, and has been related with either health or disease statuses of the host^16,17^.

Understanding host-microbe interactions in cetaceans may contribute to the identification of compromised populations, microbial markers of disease and ultimately to scientifically based population management decisions. In this sense, high throughput sequencing technologies paved the way towards implementing innovative monitoring approaches such as the environmental surveillance of the ocean ecosystem^18,19^.

There are currently twenty-one studies on the cetacean microbiota available, mostly focused on bottlenose dolphin (*Tursiops truncatus*) samples retrieved in different regions of the world, including captive and in the wild individuals^20–29^. Godoy-Vitorino *et al*.^12^ published the first report of a female striped dolphin (*Stenella coeruleoalba*) microbiome from different body sites. Other cetacean-related studies include the killer whale and humpback whale, which showed associations between skin microbiota and episodic migrations^27^.

In the present study, we assessed the oral microbiome of the 3 small cetacean species most commonly found stranded in the Atlantic Iberian coast (*S. coeruleoalba, D. delphis*, and *P. phocoena*), the latter two have never had their microbiomes characterized before. The oral cavity is a body site of easy accessibility due to its non-invasive nature, allowing a fast sampling procedure, which can be systematically used in either dead or live animals, while contributing for the preservation of sample integrity. A correlation analysis was applied to determine whether each of the analyzed cetacean species holds unique features in their microbiome profile, allowing species segregation based on their microbiome. The microbiome profiles were also evaluated (regardless of cetacean species) to identify discriminatory fingerprints according to age class, ecology of the animal or cause of death. The correlation analysis was also tested using 16S rDNA-amplicon data from a previous study focused on bottlenose dolphins^17^ to evaluate if the proposed data analysis would detect similar discriminatory patterns, using data from different experimental setups. Ultimately, we hypothesize that the correlation analysis approach used in the present work may be applied to 16S rDNA-amplicon datasets obtained with different sequencing technologies using samples from different geographic locations, facilitating data comparisons between different research studies on the microbiome composition of several cetacean species.

## Results

### Structure of the oral community of 3 species of Odontoceti cetaceans

In the present work, the standardized pipeline established by Godoy-Vitorino *et al*.^12^ was used to study the microbiome composition from the oral cavity of 3 species of Odontoceti cetaceans (Supplementary Table S1).

A total of 4430357 Miseq paired reads were filtered and merged into 2904009 high quality-filtered sequences comprising 251 bp of the V4 region of the 16S rRNA gene. After the removal of unclassified sequences, a total of 2539933 sequences were binned and classified into 1329 OTUs (Supplementary Table S2). In total, 29 bacterial phyla were identified, being distributed by 70 classes, 215 families and 483 assigned genus (Supplementary Table S2). All oral microbial communities were dominated by members belonging to the *Proteobacteria*, *Firmicutes*, *Bacteroidetes* and *Fusobacteria* phyla (Supplementary Fig. S1), which were also predominantly detected in the oral cavity of bottlenose dolphins (*Tursiops truncatus*), described by Bik *et al*.^17^.

### The microbial community from the oral cavity differs among species and at different development stages of the animal

The sampled bacterial communities were subjected to a Canonical Correspondence Analysis (CCA) to discriminate the biological and ecological factors underlying their variability (CCA is sensitive to the less abundant and unique species in the samples).

**Figure 1:**
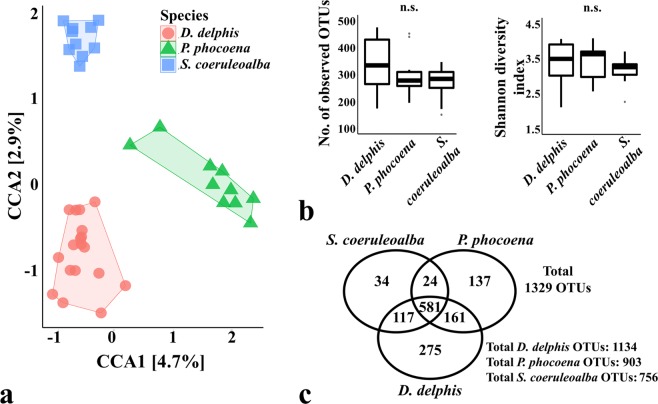
Comparison of the oral microbiomes of cetaceans according to the species of the sampled animals. Panel (a) shows the Canonical Correspondence Analysis (CCA) (p-value = 0.001) which was performed after subsampling the OTU table to even sequencing depth and at the bacterial species level, with Hellinger transformation of the abundances. Similar results were obtained when grouping the OTUs by bacterial genera. The colour frames in both CCA plots group the samples according to the constrained variable. The major contributions of the “host species” variable are shown as % in the first and second component of the CCA plot (CCA1 and CCA2, respectively). Panel (b) shows the richness and diversity measures calculated between species (as the average observed OTUs and Shannon diversity index of each sample), using the OTU table normalized by total sum scaling. Significance of these alpha-diversity metrics was tested with Kruskal-Wallis chi-squared test followed by pairwise Wilcoxon test between groups (n.s, statistically not significant). Panel c shows the number of total OTUs in each group and the number of OTUs shared between groups. *Delphinus delphis* (n = 18), *Phocoena phocoena* (n = 10) and *Stenella coeruleoalba* (n = 10).

The ordination according to the animal phylogeny (Fig. 1) grouped the samples of the same species together, clustered separately from the other two species (CCA p-value = 0.001 when considering bacterial species).

Although this clustering profile suggested the existence of interspecific differences in the microbial communities of the oral cavity, the significance was only observed for the comparison between the *P. phocoena* samples *vs*. the *D. delphis*-*S. coeruleoalba* group (CCA1 component p-value = 0.001; CCA2 component p-value = 0.224). Samples from both species *D. delphis* and *S. coeruleoalba* were in fact clustered more closely, which also reflected their closeer phylogenetic relationship: *D. delphis* and *S. coeruleoalba* belong to the family *Delphinidae* while *P. phocoena* belong to the family *Phocoenidae* (the phylogenetic trees based on the nucleotide sequence of the mitochondrial genes coding for the cytochrome c oxidase polypetide I and the cytochrome b are presented in the Supplementary Fig. S2).

Samples from *D. delphis* showed higher average number of OTUs and Shannon index values than the samples collected from *P. phocoena* and *S. coeruleoalba* (although not statistically significant), which could suggest a greater interspecific variation of the microbiota colonizing the oral cavity of these animals (Fig. 1b). The oral microbiome associated to *D. delphis*, *P. phocoena* and *S. coeruleoalba* were comprised by a total of 1134 OTUs, 903 OTUs and only 756 OTUs, respectively (Fig. 1c). From the 1329 OTUs detected in this study, the 3 species shared 581 OTUs (around 44%). Whereas the microbial community of *D. delphis* presented similar number of common OTUs with the other 2 species (117 with *S. coeruleoalba* and 161 with *P. phocoena*), the *S. coeruleoalba* and *P. phocoena* communities shared only 24 OTUs.

**Figure 2:**
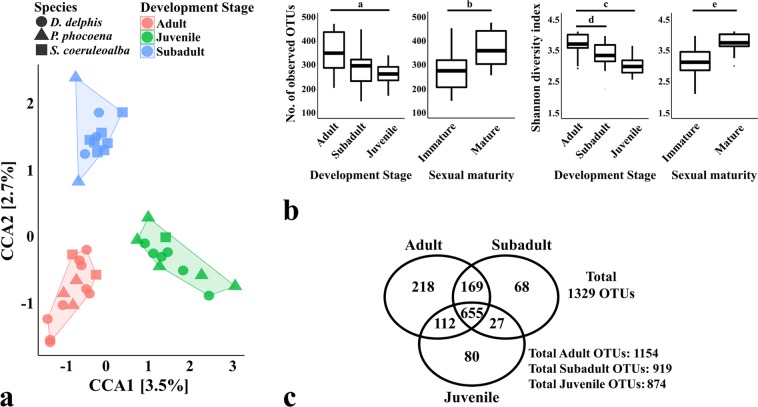
Comparison of the oral microbiomes of cetaceans according to the development stage/age class. The Canonical Correspondence Analysis (CCA) in panel (a) (p-value = 0.001) was performed after subsampling the OTU table to even sequencing depth and at the bacterial species level, with Hellinger transformation of the abundances. Similar results were obtained when grouping the OTUs by bacterial genera. The colour frames in both CCA plots group the samples according to the constrained variable and the symbols represent the species of the sampled animals: *D. delphis* (●), *P. phocoena* (▲) or *S. coeruleoalba* (■). The major contributions of the “development stage/age class” variable are shown as % in the first and second component of the CCA plot (CCA1 and CCA2, respectively). Panel (b) shows the richness and diversity measures calculated between species (as the average observed OTUs and Shannon diversity index of each sample), using the OTU table normalized by total sum scaling. Significance of these alpha-diversity metrics was tested with Kruskal-Wallis chi-squared test followed by pairwise Wilcoxon test between groups (*a*, p-value = 0.008; *b*, p-value = 0.002; *c*, p-value = 0.001; *d*, p-value = 0.002; *e*, p-value = 0.001). Panel c shows the number of total OTUs in each group and the number of OTUs shared between groups. Adult (n = 14), Subadult (n = 12) and Juvenile animals (n = 12).

A significant clustering was also observed in the CCA plot according to the development stage of the sampled individuals (Fig. 2a; CCA p-value = 0.001), in which all samples from juvenile animals, regardless of their species, were grouped separately from the more closely located groups of adult and sub-adult animals (CCA1 component p-value = 0.012; CCA2 component p-value = 0.167).

The differential clustering shown in Fig. 2a was also observed between immature and mature specimens, when constraining the CCA according to the sexual maturity of the animals (CCA p-value = 0.033, data not shown), which supports the idea of an evolving microbiome throughout the maturation and aging.

According to Fig. 2b, the composition of the microbial community from adult and mature animals was composed by a higher average number of OTUs and Shannon diversity index, when compared to the oral community of juvenile and immature animals (p-value for pairwise Wilcoxon rank tests for both alpha-diversity metrics: adult vs. juvenile <0.008; mature vs. immature <0.002).

A total of 1154 OTUs were detected in adults, 919 OTUs in sub-adult animals and 874 OTUs in juveniles (Fig. 2c), from which 655 OTUs were common among the 3 groups. Juveniles and sub-adult animals shared only 27 OTUs (3%), whereas a larger fraction was shared between each one of these two groups and adult animals (>12%).

### The composition of the oral microbial community of cetaceans is also affected by the biogeography and cause of death of the animal

**Figure 3:**
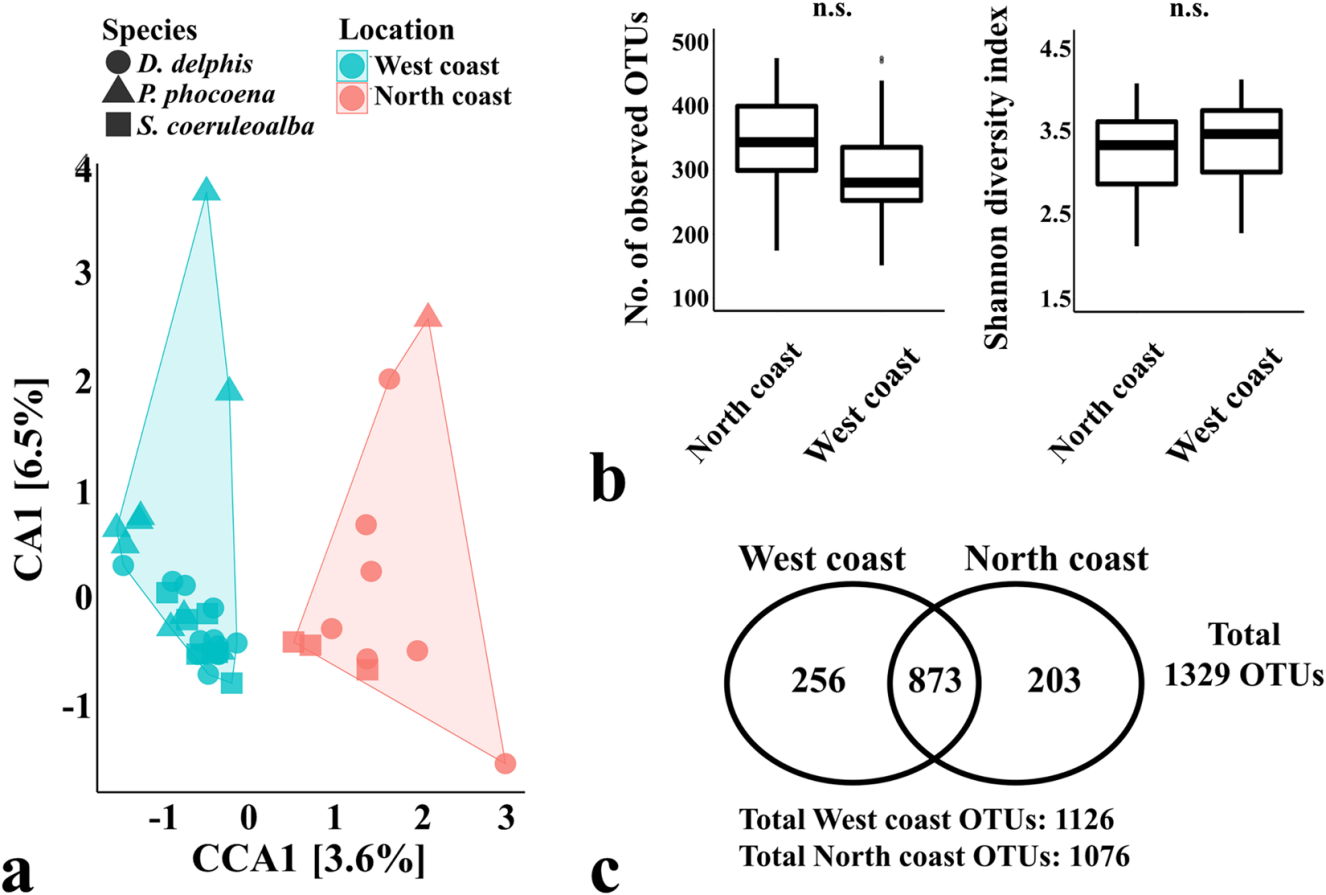
Comparison of the oral microbiomes of cetaceans according to the stranding location of the specimen. The Canonical Correspondence Analysis (CCA) in panel (a) (p-value = 0.046) was performed after subsampling the OTU table to even sequencing depth and at the bacterial species level, with Hellinger transformation of the abundances. Similar results were obtained when grouping the OTUs by bacterial genera. The colour frames in both CCA plots group the samples according to the constrained variable and the symbols represent the species of the sampled animals: *D. delphis* (●), *P. phocoena* (▲) or *S. coeruleoalba* (■). The major contributions of the “stranding location” variable are shown as % in the first and second component of the CCA plot (CCA1 and CCA2, respectively). Panel (b) shows the richness and diversity measures calculated between species (as the average observed OTUs and Shannon diversity index of each sample), using the OTU table normalized by total sum scaling. Significance of these alpha-diversity metrics was tested with Kruskal-Wallis chi-squared test followed by pairwise Wilcoxon test between groups (n.s., statistically not significant). Panel c shows the number of total OTUs in each group and the number of OTUs shared between groups. Animals sampled in the northern Atlantic Iberian coast (n = 11) and animals sampled in the western Atlantic Iberian coast (n = 27).

The CCA in Fig. 3 shows differences between samples collected from the northern and western Atlantic Iberian coast (CCA p-value = 0.046). This clustering may suggest the existence of 2 communities of cetaceans that may have a different biogeography, thus reflecting different social habits, different use of space or the utilization of different food resources available throughout the Atlantic Iberian coast. Nevertheless, the alpha-diversity metrics did not show significant differences between both locations (Fig. 3b) and they share approximately 80% of the total number of OTUs (Fig. 3c).

**Figure 4:**
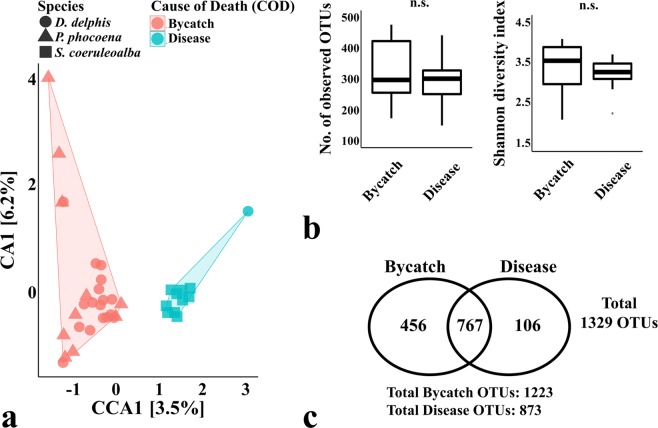
Comparison of the oral microbiomes of cetaceans according to their cause of death. The Canonical Correspondence Analysis (CCA) in panel (a) (p-value = 0.013) was performed after subsampling the OTU table to even sequencing depth and at the bacterial species level, with Hellinger transformation of the abundances. Similar results were obtained when grouping the OTUs by bacterial genera. The colour frames in both CCA plots group the samples according to the constrained variable and the symbols represent the species of the sampled animals: *D. delphis* (●), *P. phocoena* (▲) or *S. coeruleoalba* (■). The major contributions of the “cause of death” variable are shown as % in the first and second component of the CCA plot (CCA1 and CCA2, respectively). Panel (b) shows the richness and diversity measures calculated between species (as the average observed OTUs and Shannon diversity index of each sample), using the OTU table normalized by total sum scaling. Significance of these alpha-diversity metrics was tested with Kruskal-Wallis chi-squared test followed by pairwise Wilcoxon test between groups (n.s., statistically not significant). Panel c shows the number of total OTUs in each group and the number of OTUs shared between groups. Sampled animals resulting from bycatch (n = 27) and stranded diseased animals (n = 11).

The samples collected from animals due to accidental capture (bycatches) by fisheries and from stranded diseased animals were also clustered into two distinct groups (CCA p-value = 0.013), as observed in Fig. 4.

Similarly to what is observed in other mammals, there may be a tight relation between the overall health status of the cetacean and the its oral microbiome composition^30–35^. Even though all samples were derived from strandings (that resulted in death), the diseased animals seemed to comprise a less diverse oral microbiome, with a total of 873 OTUs, whereas the group of accidental captured (bycatches) totalled 1223 OTUs (Fig. 4). The health status of stranded cetaceans, which do not show signs of bycatch, is usually poor and associated with illness (pathologies and infections), or trauma. The weakened animal may end up in shallow waters, resulting in stranding and, usually, death.

### The discriminatory power of the oral microbial community is also observed in datasets of healthy cetaceans

In order to validate the discriminatory power of the variables under study in healthy cetaceans, the sequencing data from 25 animals, obtained by Bik *et al*.^17^, was also analysed in this work (Supplementary Table S3). Although composed solely by bottlenose dolphins (*T. truncatus*), the two sampled populations, from San Diego Bay (San Diego, USA) and Sarasota Bay (Florida, USA), also comprised female and male animals, at different ages (classified as stages of the development in the present study). Their oral microbial communities were also sampled with swabs and the extracted DNA was sequenced by pyrosequencing chemistry, using primers targeting the V3-V4-V5 region of the 16S rRNA gene. The OTU tables from both studies were merged to remove redundant taxa and subsampled to an even sequencing depth of 1019 sequences. Since only 54 OTUs shared the same representative sequence (Supplementary Table S4), the constrained ordinations were carried out at the bacterial genus level. From a total of 353 classified genera, 63 were detected in both studies.

Bik *et al*.^17^ did not find a clustering effect for animal age (2–51 years old) or gender, using the non-metric multidimensional scaling based on the Bray-Curtis distances of the *T. truncatus* microbial communities. Similarly, the ordination of the data obtained in the present study using Bray-Curtis distances also did not result in a clear clustering of samples according to the tested variables (data not shown).

**Figure 5:**
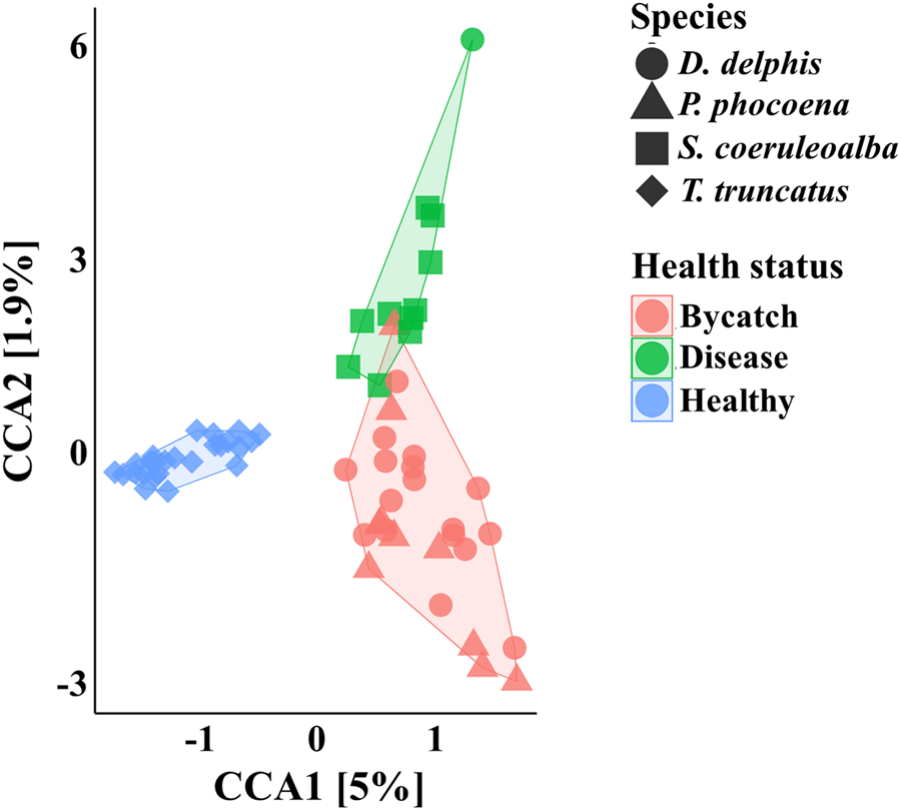
CCA of the oral microbiomes of the 4 cetacean species, according to their health status. Canonical Correspondance Analysis of the oral microbial communities of cetaceans sampled in this study and by Bik *et al*.^17^, constrained according to the health status of the sampled animals (p-value = 0.001). The color frames in the plot group the samples according to the constrained variable, comprising 3 groups: dead animals derived from bycatch (n = 27), stranded diseased animals (n = 11) and healthy animals sampled by capture and release procedure (n = 25) in Bik *et al*.^17^. Both ordinations were performed after subsampling the OTU tables to even sequencing depth of 1019 sequences and at the genus level, with Hellinger transformation of the abundances. In both panels, the species of the sampled animals are represented by symbols: *D. delphis* (●), *P. phocoena* (▲) or *S. coeruleoalba* (■) or *T. truncatus* (♦). The major contributions of the “health status” variable are shown as % in the first and second component of the CCA plot (CCA1 and CCA2, respectively).

The CCA of the oral microbiotas from both studies clustered the samples into different groups, according to the animal species (CCA1 component p-value = 0.001; CCA2 component p-value = 0.001) and their location (CCA1 component p-value = 0.001; CCA2 component p-value = 0.001), as shown in Supplementary Fig. S3. Strikingly, the CCA grouped the cetaceans into distinct groups, when constrained by the health status of the animal (Fig. 5), in which the dead animals from our study were clustered together, apart from the healthy animals sampled by Bik *et al*.^17^ (CCA1 component p-value = 0.001; CCA2 component p-value = 0.015). We do not discard the role of host-specific factors especially our samples, as most of the diseased animals were *Stenella coeruleoalba*, except for one *D. delphis*.

Significant differences in CCA clustering of the merged datasets were also maintained between adults and juveniles (CCA1 component p-value = 0.012; CCA2 component p-value = 0.005; Supplementary Fig. S3) and between mature and immature animals (CCA p-value = 0.001; CCA plot not shown).

### Commensal oral community among the 3 cetacean species sampled in this study

**Figure 6:**
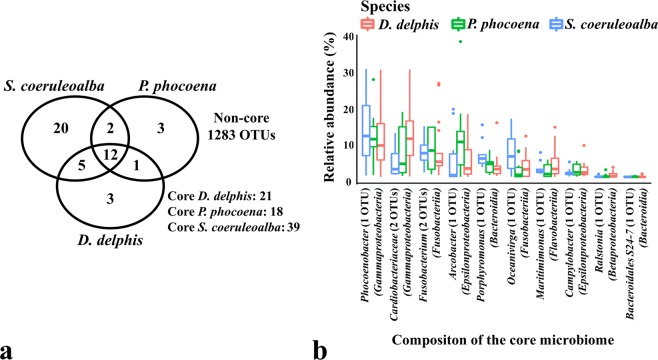
Core OTUs among the different cetacean species (**a**) and present in all 38 samples (**b**). In panel (a), the number of OTUs comprising the core microbiome of each group is represented by the OTUs present in all respective samples. In panel (b), the taxa abundance of the OTUs present in all samples is shown as the average relative frequency for each genera (or family when a genus was not assigned).

Given the different clusters obtained by CCA (Figs 1–4), we filtered the microbial profiles to identify the core community among the sampled cetaceans (Supplementary Table S5). Contrary to the total number of OTUs comprising the microbial communities of the 3 species, the number of OTUs present in all samples of each species (the species core microbiomes) was composed by a small fraction of the total OTUs (Fig. 6a), which evidenced the high heterogeneity of the samples. A similar trend was found grouping the samples by age class, sexual maturity, stranding location and cause of death (data not shown).

The core microbiome of *D. delphis*, *P. phocoena* and *S. coeruleoalba* were composed by 21, 18 and 39 OTUs, respectively (Supplementary Table S5), but their genus-level richness did not differ significantly (data not shown). Regardless of the animal species, the core microbiome of all 38 samples was composed by 12 OTUs, such as *Phocoenobacter* (OTU0), *Porphyromonas* (OTU4), *Oceanivirga* (OTU3), *Arcobacter* (OTU6) and *Fusobacterium* (OTU2, OTU266), as well as members of the genera *Campylobacter* (OTU32), *Ralstonia* (OTU83) and *Maritimimonas* (OTU16), 2 OTUs of the *Cardiobacteriaceae* family (OTU5, OTU157) and 1 OTU of the *Bacteroidales* S24-7 family (OTU244) (Fig. 6b and Supplementary Table S5). Some of these taxa have been detected in the oral cavities of other cetaceans and may represent the core symbiotic community colonizing the oral cavity of cetaceans^17,29^.

### Microbial fingerprints within the oral cavity of cetaceans

The taxa driving the observed CCA were identified by (i) a sPLS-DA to identify the major bacterial genera contributing to the separation of samples, followed by the Indicator Species Analysis of the selected genera and respective OTUs, and (ii) the linear discriminant analysis (LDA) effect size algorithm (LEfSe), as detailed in Supplementary Table S6.

**Figure 7:**
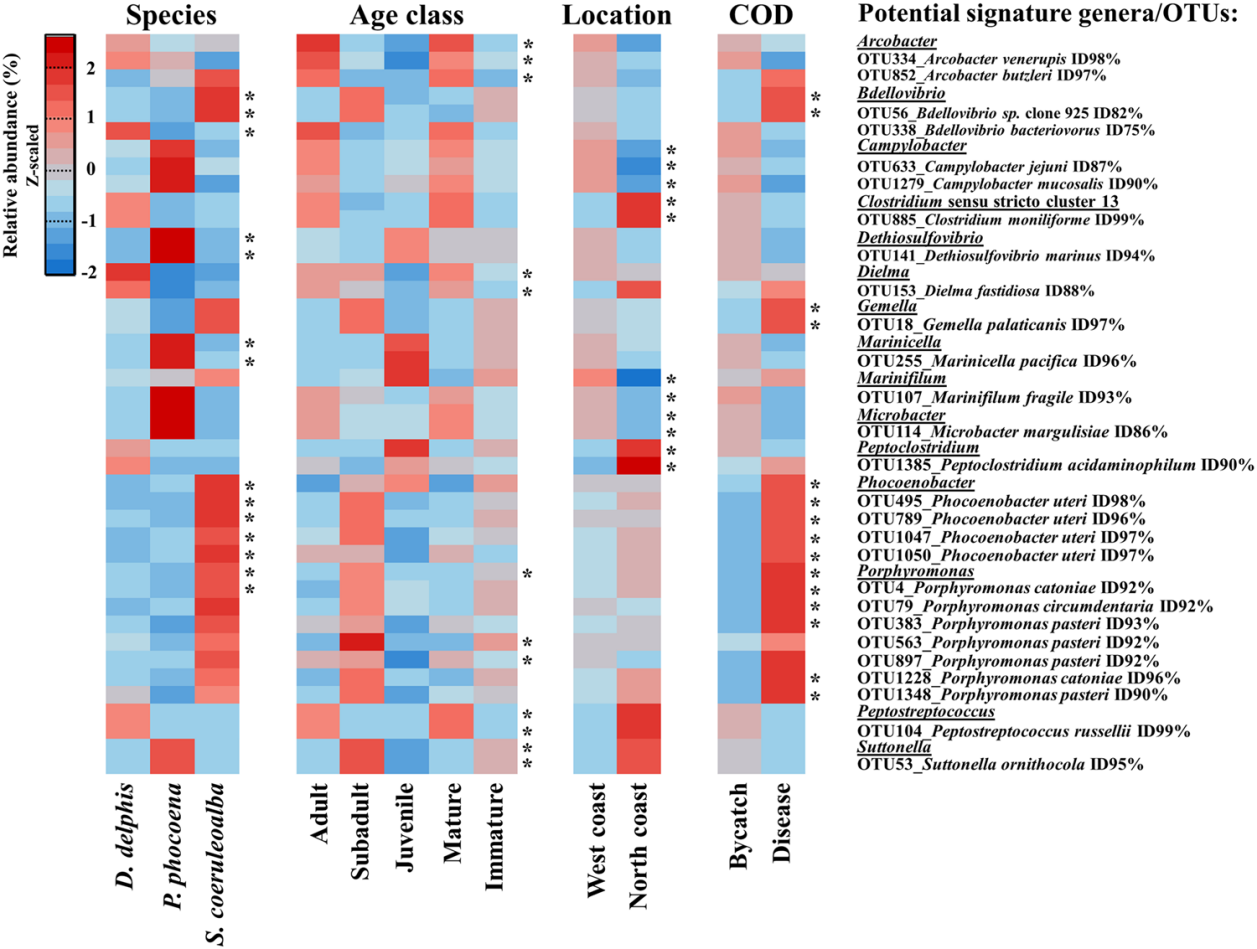
Bacterial genera and OTUs with different relative abundance between the groups of variables under study. The significance of potential microbial fingerprints (highlighted with “*”, p-value < 0.05) were identify by the Indicator Species Analysis and the linear discriminant analysis (LDA) effect size algorithm (LEfSe), detailed in Supplementary Table S6. Only the taxa showing significant differential abundance at the genus and OTU levels by both methods were considered. For representation and due to a broad range of values, the relative abundances were Z-scaled to highlight their comparisons between groups. Taxonomic validation of the representative sequences of the selected OTUs was performed with BLASTN analysis against the NCBI non-redundant nucleotide database.

Only the taxa showing statistical significance at the genus and OTU level (p-value < 0.05) in both approaches were considered potential microbial signatures of the associated groups (Fig. 7).

From a total of 21 bacterial genera and 40 OTUs that were considered potential bacterial signatures between the sampled cetacean species (Supplementary Table S6), 5 genera and 9 OTUs were detected as statistically significant by both approaches (Fig. 7). The bacterial genera *Dethiosulfovibrio* (OTU141, with a 94% nucleotide identity corresponding to the anaerobic *Dethiosulfovibrio marinus* strain WS100^36^; and *Marinicella* (OTU255, with a 96% nucleotide identity corresponding to the marine *Marinicella pacifica* strain sw153^37^) were associated to harbour porpoise samples (*P. phocoena*). Two *Bdellovibrio* OTUs (OTU56 and OTU338) with low nucleotide homology to known species (<90%) were highly represented in the clustering of common dolphins and striped dolphins (*D. delphis*-*S. coeruleoalba*). *Bdellovibrio* OTU338 was associated with the *D. delphis* group and the *Bdellovibrio* OTU56 with *S. coeruleoalba*. Moreover, *S. coeruleoalba* samples were also associated with the predominance of *Phocoenobacter* (4 OTUs, in particular with OTU495, with a 98% nucleotide homology to *Phocoenobacter uteri* strain M1063U/93) and *Porphyromonas* genera (OTU4, which was identified as a member of the core microbiome of the 3 cetaceans). However, the *Bdellovibrio* OTU56, the *Phocoenobacter* and *Porphyromonas* OTUs, in particular *Porphyromonas* OTU4, were also associated with diseased animals (as shown in Fig. 7), which suggested that the abundance and balance of these taxa in the community may be determinant to their pathogenic potential. The genus *Gemella* and the *Gemella* OTU18 were also associated with the group of diseased animals of the present study.

To identify taxa that could discriminate healthy from diseased animals, the Indicator Species Analysis and LEfSe were applied to the combined CCA including the animals from this study and those from Bik *et al*.^17^, as observed in Fig. 5. When clustering was performed according to species or location (Supplementary Fig. S3), the samples associated to the groups of *T. truncatus* in USA populations (Sarosota Bay and San Diego Bay) were the same samples leading to the healthy specimens’ group on the CCA (Fig. 5). After filtering the bacterial genera and OTUs contributing to the clustering according to these 3 variables (species, location and health status), the the OTU79, OTU383, OTU1228, OTU1348 from *Porphyromonas* genus were identified as potential signature of the diseased animals (p-value < 0.05 by the Indicator Species Analysis and LEfSe). Moreover, the genera *Bradyrhizobium* (OTU419), *Escherichia* (OTU130) and *Pseudomonas* (OTU69, OTU155, OTU168) were also considered as potential indicators of disease when using the merged datasets. Furthermore, they were not detected in the oral microbiome of healthy *T. truncatus*.

The clustering of the healthy animals was specifically associated with the presence of 4 OTUs of *Desulfobacterium* spp., 2 OTUs of *Tannerella* spp. and 1 OTU of *Thalassobius* spp., none of them present in the oral communities of the diseased animals sampled in this study. The genera *Marinifilum* (17 OTUs), *Maritimimonas* (10 OTUs), *Microbacter* (4 OTUs)*, Sphaerochaeta* (2 OTUs) and *Thiothrix* (4 OTUs) were also considered as potential signature taxa of the non-diseased status of the studied cetaceans, by being present in healthy or accidentally captured animals and contributing with several OTUs to the CCA clustering (Fig. 5 and Supplementary Table S7). Nevertheless, due to the low number of common OTUs and bacterial genera between both studies, the significance of potential signature taxa associated with a healthy *vs*. diseased status may have been biased, by the absence of several taxa in one of the tested groups.

A total of 20 genera and 38 OTUs were considered potential bacterial signatures for the different age classes (development stage and sexual maturity) of the sampled animals (Supplementary Table S6), from which 5 genera and 7 OTUs were common for both methods used to identify the taxa driving the observed CCA (Fig. 7). The bacterial genera *Arcobacter* (OTU334 with a 98% nucleotide homology to *Arcobacter venerupis* strain CECT7836T and OTU852 with a 97% homology to *Arcobacter butzleri* strain NCTC12481), *Dielma* (OTU153 with a 88% homology to *Dielma fastidiosa* strain JC13) and *Peptostreptococcus* (OTU104 with a 99% homology to *Peptostreptococcus russellii* strain ING2-D1D) were associated with adult/mature animals. Members of the genera *Porphyromonas* (OTU563 and OTU897, both with a 92% homology to *Porphyromonas pasteri* strain KUFDS01) and *Suttonella* (OTU53 with a 95% homology to *Suttonella ornithocola* strain B6/99/2) were associated with animals in the subadult stage.

The separation according to the location of the animals was associated to 22 genera and 27 OTUs (Supplementary Table S6), with a significant overrepresentation of the genera *Campylobacter* (OTU663 and OTU1279 with <90% homology to known species), *Marinifilum* (OTU107 with 93% homology to *Marinifilum fragile* strain JC2469), *Microbacter* (OTU114 with 86% homology to *Microbacter margulisiae* strain ADRI) in the animals of the western Atlantic Iberian coast, whereas members of *Clostridium sensu stricto* cluster 1 (OTU885 with 99% homology to *Clostridium moniliforme* strain HYN0057) and *Peptoclostridium* (OTU1385 with 90% homology to *Peptoclostridium acidaminophilum* strain a1-2) were associated to the animals of the northern Atlantic Iberian coast (Fig. 7).

## Discussion

Metagenomic studies in mammals have been mainly focused on the gut-related microbiome of terrestrial animals. Variations in the microbial community structure have been associated with the (i) host phylogeny, in which different species co-inhabiting the same niche have shown species- or population-specific gut microbiomes^38–40^, (ii) host age and development stage^41–43^, host gender^44,45^, host biogeography and social habits^43,46–51^. Little is known about the inter- and/or intraspecific variability of the microbiomes of cetaceans. Due to the high heterogeneity of the samples from the 38 Odontoceti used in the present study, clustering patterns of their oral microbiomes were not clearly observed with ordinations based on Bray-Curtis, as also reported by Bik *et al*.^17^. The ecology of these animals has been described to drive genetic differentiation between populations of cetaceans inhabiting the Atlantic Ocean, the North Sea and the Mediterranean Sea^52–55^, which could also contribute to different predispositions for the oral colonization of specific taxa. Apprill *et al*.^27^ compared the skin microbiome of humpback whales from subtropical waters (near Hawaiian Islands) and Alaska waters, and described the geographic distribution of the animals as a shaping factor for the differences observed in the structure of these microbial communities, exposed to different conditions of the surrounding environment.

The heterogeneity of the oral samples in the present study was evidenced by the variation of the alpha-diversity values and by the low number of core OTUs predicted for each species (1.9%, 1.9% and 5.2% of the total OTUs of *D. delphis*, *P. phocoena* and *S. coeruleoalba*, respectively). This heterogeneity was also observed in Bik *et al*.^17^, since only 2 OTUs were present in all oral samples of *T. truncatus*. According to Apprill and co-workers^26^, the drone-captured blowhole samples from humpback whales *Megaptera novaeangiliae* revealed a microbiome composed by 25 core OTUs and only 2 bacterial genera comprised the core skin microbiome of this whale species^27^. In contrast, Erwin and co-workers reported 147 and 155 OTUs composing the core gut microbiome of the pygmy (*Kogia breviceps*) and dwarf (*Kogia sima*) sperm whales^28^. These reports evidence a greater variability associated with the microbiome of body sites exposed to the external environment and their concomitant sampling methods, in contrast with the microbiome of internal body sites (e.g. gut), which might be prone to a lower degree of variation regarding their microbial richness. In this respect, this initial analysis could suggest that evaluating the oral cavity microbiota composition may not constitute the best method to detect overall variations in the health status of cetacean populations, unless a large number of samples is available. However, these are pioneering results, which can be used in future non-invasive biomonitoring studies as a comparison baseline from which health changes or deviations will be evaluated.

The core microbiome of all 38 samples analyzed in this study is composed by 12 OTUs, most of them related to human pathogens. *Pasteurellaceae* members, such as the *Phocoenobacter* OTU0, can be commensals. This OTU showed a 99% nucleotide identity with *Phocoenobacter uteri*, a species that was related to septicaemia in marine mammals^56,57^. However this species was also found in apparently healthy individuals^17^. *Porphyromonas* OTU4 showed a 92% nucleotide homology to *Porphyromonas catoniae* strain ATCC 51270, whose abundance was associated with a caries-free oral status in humans^58^, and was detected in the oral cavity of the healthy *T. truncatus* reported by Bik *et al*.^17^. Similarly, even though commensal *Fusobacterium* strains have been associated to several pathologies in humans (e.g. inflammatory bowel disease, oral carcinoma)^59,60^ and in a diseased striped dolphin (Godoy-Vitorino, 2017), 16 *Fusobacterium* OTUs were detected in the oral microbiomes of healthy bottlenose dolphins^17^. *Campylobacteraceae* genera, such as *Arcobacter* and *Campylobacter*, have been associated with human and animal illness^61,62^ but have also been found in marine sediments^63^ as well as in the dental plaque of non-diseased captive delphinids^29^.

*Cardiobacteriaceae* members have been associated with bacteremia and wound infections in humans^64^. They were detected in the blowhole, tongue and oral cavity of the dolphin reported by Godoy-Vitorino *et al*.^12^, as indicators of sepsis. Nevertheless, 51 OTUs belonging to the *Cardiobacteriaceae* family were detected in oral cavity of healthy bottlenose dolphins^17^. The *Bacteroidales* S24-7 family (*Candidatus Homeothermaceae*) has been predominantly found in the gut of homeothermic animals (e.g. human, mice and other rodents, koala, pig) and are described as mainly carbohydrate-fermentative and microaerophilic or anaerobic bacteria^65^.The ubiquitous presence of these 12 OTUs in all 38 samples (Fig. 6 and Supplementary Table S5), regardless animal species, age class, stranding location or the animal health status, is evidence that these bacteria may show low virulence levels in healthy animals, whose pathogenicity may be only potentiated in immunocompromised individuals. The identification of a commensal microbiota catalogue will be the cornerstone to set a baseline microbial profile of the oral cavity and more accurately determine harmful variations in the microbiome composition.

In the present study, the analysis of the oral microbiomes of cetaceans with a Canonical Constrained Analysis showed that the phylogeny and ecology of these animals shaped the microbial community structure of the oral cavity. The major variables driving the separation of the communities were the animal species and the development stage, which explained approximately 8% and 6% of the CCA variation, as shown in the axis CCA1 and CCA2 components of the CCA plots (Figs 1 and 2).

The evidenced divergence between the microbial communities according to the animal species (Fig. 1) may reflect their different use of the marine environment (striped dolphins are mostly found offshore whereas porpoises near the coast). The association of specimens from *S. coeruleoalba* and *D. delphis* has been also reported in the Mediterranean sea and in other areas of the Atlantic ocean^66,67^. The occurrence of mixed groups of *coeruleoalba* and *D. delphis* may contribute to a more similar microbiota between animals and attenuates interspecific differences, mainly because individuals share the same areas, similar food resources, they may physically interact, and they are exposed to the same environmental chemicals. To mitigate eventual bias regarding the inter-species analysis of bacterial richness and diversity derived from different species group size (common dolphin group includes18 animals, in comparison to 10 harbour porpoises and 10 striped dolphins), the same clustering analysis was performed after normalizing the “Species” groups to 10 animals. Supplementary Fig. S5 shows that the number of sampled animals did not affect the CCA clustering pattern (p-value = 0.001 in all panels), neither resulted in statistically significant differences in the comparisons of the average number of OTUs and Shannon index values between the difference cetaceans species. The variation in the degree of data dispersion was the only effect observed in Supplementary Fig. S5b,c, which derived from the different contribution in OTUs that each animal might bring to the combined analysis, as a result of the biological variation inherent to the type of samples. A similar trend was observed in the sample number normalizations carried out for the other groups of variables under study (data not shown).

Reports of delphinid schools predominantly composed by animals of similar ages are not uncommon and support the clustering observed^68,69^. For instance, in the western Pacific, *S. coeruleoalba* schools have been described to be composed either by juveniles, adults or by both types of animals^68^. Juvenile schools may inhabit coastal waters, which usually offer more protection from predators. Younger members (calves) can remain in adult schools until completing 2 years of age and then join juvenile schools, until reaching adulthood. After reaching sexual maturity, sub-adult animals may join breeding or non-breeding adult schools. Breeding schools are usually comprised by sexually mature adult females and may also include adult males. Sub-adult and adult males in breeding schools may leave, after most females are pregnant. Thus, the breeding school evolves into a non-breeding adult school, which will later include their calves^68^. Age-related differences in the gut microbiome were also described for other mammals such as the ring-tailed lemurs, Zucker rat and the Australian fur seal^41–43^. Although common and striped dolphins mixed groups can be found at times, the separation according to host species was expected. This oral microbiome community separation is, in fact, a good indication of the applicability of such data in the future to assess health population changes within species. The oral microbiome community separation according to development stage is even more important, since it indicates that deviations (health imbalances) may be connected to and affect by the dynamics of population structure.

The statistically significant CCA clustering according to stranding location might be biased by the animals’ ability to travel large distances along the coast or away from the coast (the microbial profile of the water or of the prey that could associate the microbial profiles and the potential signature genera to the different areas is not known). Therefore, microbial profiles of the main prey for each cetacean species, as well as microbial profiles of seawater in known cetacean occurrence hotspot areas, are needed. A significant CCA clustering of the samples based on bacterial genera was not obtained when the gender of the animals was used as biological variable (female *vs*. male animals). The lack of clustering according to host gender was expected considering that the analysed cetaceans species are composed of highly social individuals (less so for harbour porpoises) often forming mixed-sex groups. Significant differences were only detected when the microbial communities were compared at the family level (CCA p-value = 0.031). This is one of the more important results of this study, considering the severe conservation status of the porpoise population in Portugal. Microbiome fingerprinting for Phocoenids (or *Phocoena phocoena*, the only Phocoenidae representative in the North Atlantic) will prove very valuable in the future since the population is likely to undergo local extinction within decades^9^. If the population continues to decline, monitoring the evolution of the Phocoenidae health status will be critical to identifying impacts, sources and solutions that may contribute to halting the population decline.

The CCA approach presented in this study also organized the pyrosequencing datasets from Bik *et al*.^17^ into different groups, when constrained according to the location (p-value = 0.001, using bacterial genus or OTUs), development stage and sexual maturity (p-value using bacterial genus = 0.205; p-value using OTUs = 0.038) of the animals (Supplementary Fig. S4). Furthermore, when our data was merged with the data from Bik *et al*.^17^, similar CCA clustering patterns were observed (despite the low number OTUs common to both studies), in particular regarding the development stage of the animals (p-value = 0.001; Supplementary Fig. S3), in which the samples remained ordered in 3 groups of adult, sub-adult and juvenile animals. Therefore, the approach used may facilitate data comparisons between different research studies on the microbiome composition of several cetacean populations and species.

Moreover, this study identifies a total of 15 bacterial genera and 27 species to be further explored as microbiota fingerprints within the oral cavity of cetaceans.

Therefore, the present study contributes to setting the proper knowledge ground to (i) develop fast bio-monitoring molecular diagnostic assays and tools for microbiome illnesses to predict outbreaks in these populations, as well as to (ii) develop epidemiological models based on high-throughput approaches to assess population health status of coastal cetacean species, and (iii) to identifying impacts, their probable causes, and propose solutions towards marine conservation by using cetacean microbiomes as indicators of marine ecosystem health. Furthermore, the proposed microbiome profiling approach of the oral cavity of cetaceans may become a valuable tool in emergency scenarios like in massive stranding events. In fact, sample collection for microbiome profiling is a fast and straight forward procedure and it will allow to rapidly retrieve important information of large numbers of stranded individuals (e.g. health status, development stage), without resorting to laborious necropsy procedures.

## Methods

### Sample collection

The samples analysed in this work were obtained in collaboration with the Portuguese Marine Animal Stranding Network, which is coordinated by the Instituto para a Conservação da Natureza e Florestas from Portugal (http://www.icnf.pt/portal/icnf), in cooperation with the Portuguese Wildlife Society (SPVS), and by the Spanish Coordinadora para o Estudo dos Mamíferos Mariños (CEMMA), which is responsible for the marine mammal stranding network in Galician waters. All sampled animals were found stranded along the coast. In total, 38 cetaceans (some recently dead and others still alive), were assessed along the northern (11 specimens) and western (27 specimens) Atlantic Iberian coast, by the Marine Animal Stranding Network teams in Portugal and by CEMMA in Galicia, Spain. In the case of the stranded animals found initially alive on the beach, refloating was not a possibility due to their critical clinical state, and despite the efforts of the rescuing teams, animals died before reaching the rehabilitation centre.

Samples from the oral cavity were collected by swabbing the gingival sulcus of the lower and upper jaws with sterile nylon fibber swabs (FLOQSwab^TM^, Copan), according to the procedure established previously in Godoy-Vitorino *et al*.^12^, from those animals that had died during rescuing or within less than 24 h prior to sampling (estimated from *rigor mortis* and organ temperature). Additional information regarding gender, development stage and sexual maturity (using the total length as a proxy for age class), occurrence of gross pathologies, and cause of death of the specimens was registered when possible during necropsy procedures^70,71^. Briefly, the sampled animals belonged to three different species of Odontoceti cetaceans, including 18 common dolphins (*Delphinus delphis*), 10 striped dolphins (*Stenella coeruleoalba*) and 10 harbour porpoises (*Phocoena phocoena*). After having been found stranded on the coast, the animals’ necropsies indicated that they had either been incidentally captured in fisheries (27 occurrences as bycatch; animals showing normal body condition and typical cuts caused by fishing gear) or that they had died due to disease (11 occurrences; animals showing emaciation, thin blubber layer, empty stomachs, gross lesions most frequently indicating forms of pneumonia and hepatitis, and high parasite intensity). These cetaceans were classified according to their development stages (14 adults, 12 sub-adults and 12 juveniles) and to their sexual maturity (25 immature and 13 mature), from which 25 were females and 13 males (Supplementary Table S1).

### Genomic DNA extraction, PCR amplifications and sequencing

The genomic DNA acquired by the oral cavity swabs, was extracted using the PureLink^TM^ Genomic DNA Mini Kit, according to the manufacturer’s instructions. PCR amplification of the 16S rRNA gene targeting targeting the hypervariable region V4 (forward primer: 5′ GTGCCAGCMGCCGCGGTAA 3′; reverse primer: 5′ GGACTACHVGGGTWTCTAATCC 3′), according to Kozich and co-workers^72^. The generated amplicons covered a region of 251 bp. The DNA was processed according to Illumina instructions to generate Nextera XT paired-end libraries (2 × 250 bp).

### Sequence processing and data analysis

Read pairs were trimmed with Sickle, including a minimum Phred score of 30 and a sliding window of 10% of the read length^73^, to remove adapter and primers sequences, as well as, nucleotides corresponding to low quality base calls. Read pairs were overlapped with Vsearch v2.3.2^74^ and prepared with strict quality and size filtering (minimum length of overlap between reads = 20 bp, minimum length of the merged sequence = 200 bp, maximum expected error of 0.5, maximum number of different bases in the overlap = 2) into uniform error-free sequences. Vsearch^74^ was used for the de-replication, removal of chimeric sequences and clustering with an identity threshold of 97%. The taxonomic classification was assigned with the SILVA ribosomal RNA gene database, version 128^75^, by using the non-redundant representative set of reference sequences clustered at 97% nucleotide identity. Before the analysis of the microbial profiles, the unclassified sequences and low frequency counts (singletons) were removed from the operational taxonomic unit (OTU) table.

The alpha-diversity metrics (richness - as number of OTUs and Shannon diversity index) were estimated with the *amp_alphadiv* function available in the ampvis2 package version 2.3.11^76^ from the “R” software, and the differences observed between groups of samples was tested with the Kruskal-Wallis chi-squared test, followed by pairwise Wilcoxon test between groups (*kruskal.test* and *pairwise.wilcox.test* functions).

The analysis of the community structure between samples was carried out after performing a total sum scaling normalisation (transforming abundances into relative frequency) and rarification of the OTU tables to an even sampling depth of 36760 sequences per sample, thus eliminating any bias due to differing sampling depth during the sequencing process^77^. Constrained canonical analyses (CCA) were performed according to each variable of the metadata collected, by using the *cca* and *anova.cca* functions from the vegan package version 2.5.2^78^, with 999 permutation testing. The comparisons of the microbial communities by CCA were performed at the species, genus and family level, using OTU tables with a square root transformation of the relative abundance (Hellinger transformation performed with the *decostand* function) to reduce the range of the data and to make it suitable for analysis by linear methods^79,80^. Representation of the CCA plot was performed with the *amp_ordinate* function from the ampvis2 package. The core microbiome and shared OTUs between groups of samples were estimated with the compute_core_microbiome.py and shared_phylotypes.py scripts from QIIME version 1.9.1^81^. BLASTN analysis of the representative sequences of the OTUs was performed against the NCBI non-redundant nucleotide database to identify the closest homologs of relevant taxa^82^.

### Comparison with sequencing data available for healthy cetaceans

High-throughput sequencing data regarding the oral microbiome of healthy bottlenose dolphins (*Tursiops truncatus*) was previously published by Bik *et al*.^17^. The DNA from these *T. truncatus* samples were acquired from oral swabs and sequenced with the 454 Life Sciences Genome Sequencer FLX Titanium platform, using primers targeting the V3-V4-V5 region of the 16S rRNA gene.

In order to assess if the biological and ecological variables under study could underlie variations in the microbiome of the oral cavity of healthy cetaceans, the approach used in the current work was also applied to the data from 25 animals reported by Bik *et al*.^17^. The selected sequence data (identified in Supplementary Table S3 by the SRA accession numbers) derived from two populations inhabiting in the San Diego Bay, San Diego (16 animals) and Sarasota Bay, Florida (9 animals), in the USA. They also included specimens from different gender (13 female and 12 male animals), at different stages of the development (14 adult and 11 juvenile animals) and sexual maturity (11 mature and 14 immature animals).

Initially, BLASTN was used to assess the homology between the representative sequences of the OTUs from the current study and the representative sequences available for the OTUs of Bik *et al*.^17^. Common OTUs between studies were identified when (i) 100% nucleotide identity was observed within the 251 bp comprising the V4 region of the 16S rRNA gene, targeted in the amplicon sequencing approach of the present study; (ii) only 1 blast hit with 100% nucleotide identity was available from the data of Bik *et al*.^17^. Afterwards, the OTU tables from both studies were merged as a.csv file, the duplicated common OTUs were manually removed and the final merged OTU table was converted to.biom format using the BIOM project tools^83^. The CCA and sPLS-DA were performed as described before, at the genus level and using the subsampled tables with 1019 sequences, due to the lower sequencing depth that resulted from the pyrosequencing chemistry used in the Bik *et al*. study^17^.

### Identification of discriminatory bacterial fingerprints according to animal species, age class, ecology of the animal, or cause of death

The potential signature bacterial genera contributing to the clustering of samples observed in the CCA plots were assessed by two approaches: (i) a supervised Partial Least Squares Discriminant Analysis (sPLS-DA) followed by an Indicator Species Analysis; (ii) the linear discriminant analysis (LDA) effect size algorithm (LEfSe).

The sPLS-DA was carried out with the *plsda* and *tune.splsda* functions from the mixOmics package version 6.3.1^84^ to identify the major bacterial taxa underlying the CCA profile according to the studied variables^85^. In the sPLS-DA plots, a confidence level of 95% was used to draw the confidence ellipses and highlight the sample groups obtained. The significance of the differential abundance of relevant taxa was validated by an Indicator Species Analysis^86^ coupled to a Monte Carlo significance test with 999 permutations, by using the *indval* function from the labdsv package v1.8^87^ and the original OTU table, transformed with total sum scaling. The association of each taxon to a specific metadata variable was measured by the *indval* index, which ranges from a minimum of 0 (not associated) to a maximum of 1 (good indicator), depending on the taxon abundance and fidelity (relative frequency) to a particular group. A bacterial genus was considered a potential signature taxon if the indicator analysis showed (i) a p-value < 0.05, (ii) a relative frequency associated to the grouping variable of at least 0.5, (iii) an *indval* index of at least 0.5 associated to the grouping variable, and if iv) any of the OTUs related to the potential indicator genus showed a p-value < 0.05 in the indicator analysis of the original OTU table.

The LEfSe from the Microbiomeanalyst web-tool^88^ was carried out with the original OTU table, transformed with total sum scaling, to complement and validate the previous approach. This approach involves the non-parametric factorial Kruskal-Wallis sum-rank test to identify taxa with significant differential abundances, according the grouping variables of interest, followed by Linear Discriminant Analysis (LDA) to estimate the effect size of each differentially abundant taxa^89^. The threshold of the LDA score to identify the major bacterial taxa driving the clustering of the samples was set to 2, as described in previous studies^90–93^.

Only the bacterial taxa showing significant differential abundance at the genus and OTU levels by both methods were considered as potential microbial signatures for the respective grouping variable. The differential abundance was represented by the *heatmap.2* function from the “R” software. For representation and due to a broad range of relative abundances, the values were Z-scaled (standard transformation from the function: z = (x − mean)/standard deviation) to highlight their comparisons between groups.

## Supplementary information

Supplementary

Supplementary Dataset 1

## Data Availability

The sequences datasets supporting the conclusions of this article are available in NCBI, deposited with the BioProject database ID PRJNA494623, associated with the SRA accession numbers ranging from SRR7963801 to SRR7963838. The essential data generated and analysed during this study is included in the Supplementary Dataset. The datasets of the *T. truncatus* animals analyzed during the current study, which were published by Bik *et al*.^17^, are available in NCBI (see Supplementary, Table S3 for the corresponding Sequence Read Archive accession) and as Supplementary Material of the corresponding publication (10.1038/ncomms10516).
