# Supplementary material for: Microbiota fingerprints within the oral cavity of cetaceans as indicators for population biomonitoring
Source: Sci Rep. 2019 Sep 23;9:13679. doi: 10.1038/s41598-019-50139-7 (PMC6757053; doi:10.1038/s41598-019-50139-7)
Supplement: Supplementary file 1 — Supplementary [file 41598_2019_50139_MOESM1_ESM.pdf]

Supplementary figures

Microbiota fingerprints within the oral cavity of cetaceans as indicators for population biomonitoring

Pedro Soares-Castro[a], Helena Araújo-Rodrigues[a], Filipa Godoy-Vitorino[b], Marisa Ferreira[a,c], Pablo Covelo[d], Alfredo López[d,e], José Vingada[c,e], Catarina Eira[c,e], Pedro Miguel Santos[a*]

[a] *Department of Biology and Centre for Molecular and Environmental Biology (CBMA), University of Minho, Campus de Gualtar, 4710-087 Braga, Portugal*

[b] *University of Puerto Rico, School of Medicine, Department of Microbiology and Medical Zoology, Microbial Ecology and Genomics Lab, GPO Box 365067, San Juan, Puerto Rico 00936-5067, USA*

[c] *Portuguese Wildlife Society (SPVS), Quiaios, Field Station, Apartado 16 EC Quiaios, 3081-101 Figueira da Foz, Portugal*

[d] *Coordinadora para o Estudo dos Mamíferos Mariños (CEMMA), P.O. Box 15, 36380 Pontevedra, Gondomar, Spain*

[e] *Department of Biology and CESAM, University of Aveiro, 3810-193, Aveiro, Portugal*

* Corresponding author: Pedro M. Santos, CBMA – Centre of Molecular and Environmental Biology, University of Minho, Campus de Gualtar, 4710-057 Braga, Portugal, +351253601515, psantos@bio.uminho.pt

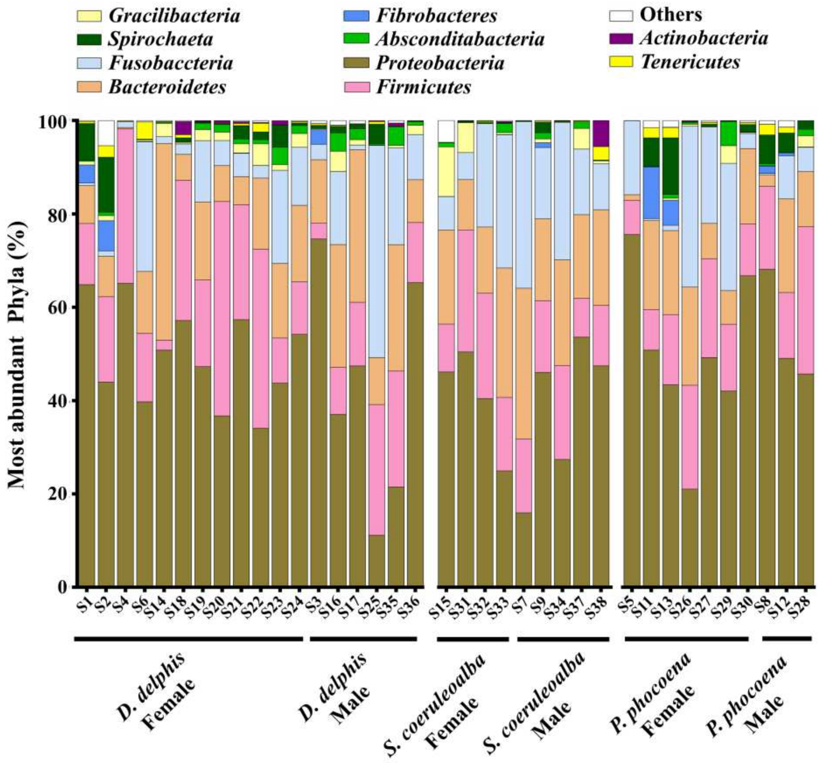

Fig. S1. Taxonomic distribution of the most abundant OTUs at the phylum level (> 5% of relative abundance across samples) in the oral cavity of the common dolphin *Delphinus delphis*, striped dolphin *Stenella coeruleoalba* and harbour porpoise *Phocoena phocoena.*

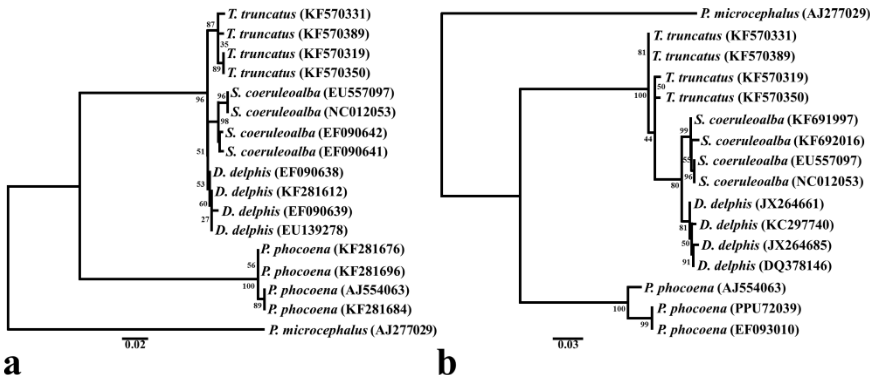

Fig. S2. Phylogeny of the common dolphin *Delphinus delphis*, striped dolphin *Stenella coeruleoalba*, harbour porpoise *Phocoena phocoena* and bottlenose dolphin *Tursiops truncatus*, based on the nucleotide sequence of their mitochondrial genes (A) *coi* and (B) *cytb*, coding for the cytochrome c oxidase polypetide I and the cytochrome b, respectively. The alignment was performed with MAFFT version 7 (Katoh and Standley, 2013) and the maximum likelihood phylogenetic midpoint rooted trees were generated by using PhyML version 3.0 (Guindon *et al*., 2010), with 1000 bootstrap sets, the GTR (General Time Reversible) as the best-fit model of nucleotide substitution (evaluated by the W-IQ-Tree tool (Trifinopoulos *et al*., 2016), kappa estimated, 4 substitution rate categories, gamma distribution parameter estimated, BIONJ starting tree, with optimization of topology, branch lengths and rate parameter. The DNA sequences used to create the tree were obtained from the Genbank database (*coi* gene: EF090638, EF090639, KF281612, EU139278 for *D. delphis*; EF090641, EF090642, EU557097, NC012053 for *S. coeruleoalba*; AJ554063, KF281686, KF281684, KF281676 for *P. phocoena*; KF570331, KF570319, KF570389, KF570350 for *T. truncatus*; *cytb* gene: JX264661, JX264685, KC297740, DQ378146 for *D. delphis*; KF691997, KF692016, EU557097, NC012053 for *S. coeruleoalba*; AJ554063, PPU72039, EF093010 for *P. phocoena*; KF570331, KF570319, KF570389, KF570350 for *T. truncatus*). The *coi* and *cytb* sequences of the sperm whale *Physeter microcephalus* (accession AJ277029) were used as an outgroup. Bootstrap values presented as percentage are shown in the tree branches.

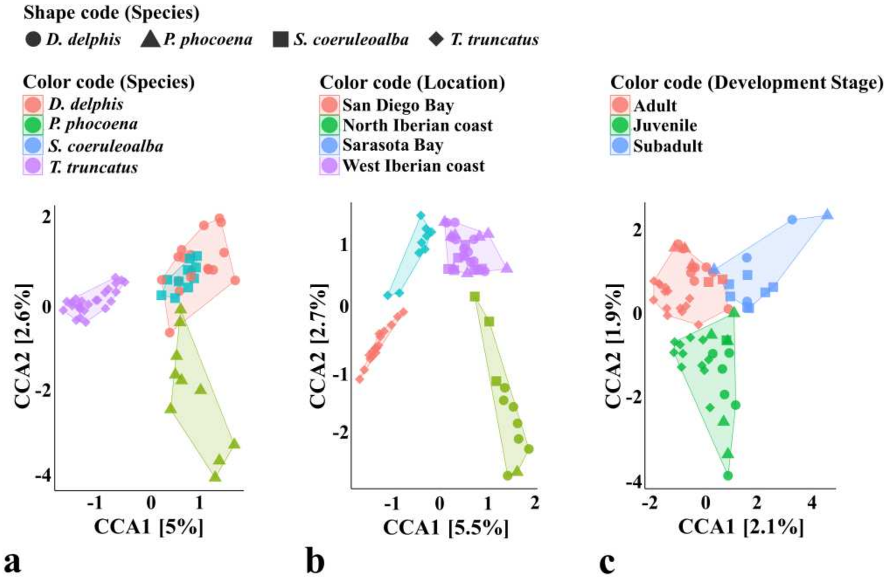

Fig. S3. Canonical Correspondance Analysis (CCA) of the oral microbial communities of cetaceans sampled in this study and by Bik *et al.* (2016), constrained according to (A) the species (p-value = 0.001) of the sampled animal, (B) the location (p-value = 0.001) and (C) the development stage of the specimen (p-value = 0.001). The colour frames in the CCA plots group the samples according to the constrained variable. The constrained ordination in panel A comprises 3 groups: *D. delphis* and *S. coeruleoalba* (n = 28), *P. phocoena* (n = 10) and *T. truncatus* (n = 25). The constrained ordination in panel B comprises 4 groups: animals sampled in this study in the northern Atlantic Iberian coast (n = 11) and in the western Atlantic Iberian coast (n = 27), as well as animals sampled by Bik *et al.* (2016) in the Sarasota Bay, Florida (n = 9) and in the San Diego Bay (n = 16), in the USA. The constrained ordination in panel C comprises 3 groups: adult (n = 28), subadult (n = 12) and juvenile animals (n = 23). All ordinations were performed after subsampling the OTU tables to even sequencing depth of 1019 sequences and at the genus level, with Hellinger transformation of the abundances. In both panels, the species of the sampled animals are represented by symbols: *D. delphis* (●), *P. phocoena* (▲) or *S. coeruleoalba* (■) or *T. truncatus* (♦).

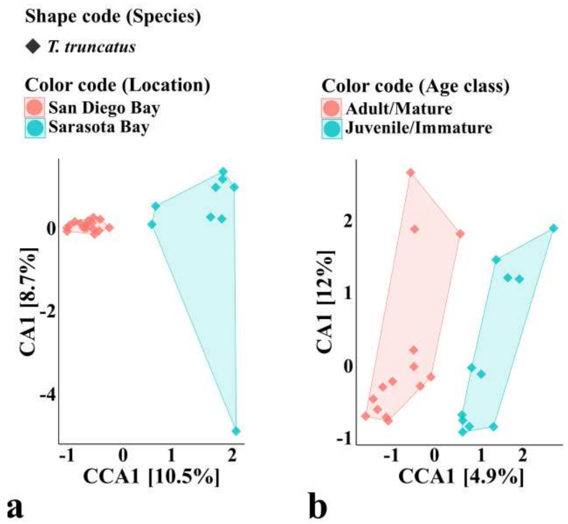

Fig. S4. Canonical Correspondance Analysis (CCA) of the oral microbial communities of cetaceans sampled by Bik *et al.* (2016), constrained according to (A) the location (p-value = 0.001) of the sampled animal, (B) the development stage/sexual maturity (p-value = 0.038). The colour frames in the CCA plots group the samples according to the constrained variable. The constrained ordination in panel A comprises 2 groups: animals sampled in the Sarasota Bay, Florida (n = 9) and in the San Diego Bay (n = 16), in the USA. The constrained ordination in panel B comprises 2 groups: adult or mature (n = 14) and juvenile or immature animals (n = 11). The ordinations were performed after subsampling the OTU tables to even sequencing depth of 1019 sequences and at the genus level, with Hellinger transformation of the abundances. In both panels, the species of the sampled animals is represented by symbols: *T. truncatus* (♦).

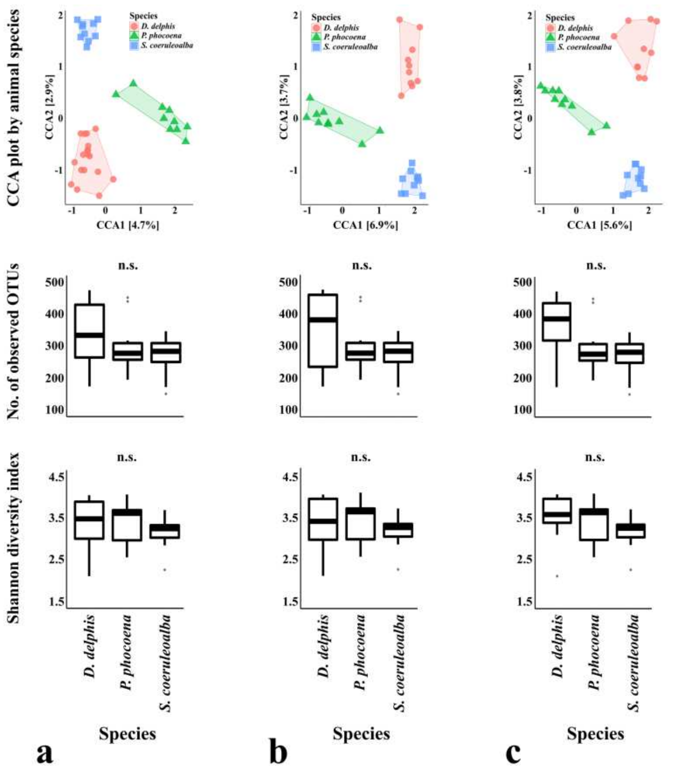

Fig. S5. Comparison of the oral microbiomes of cetaceans according to the animal species, after normalizing the number of sampled animals. Panel a shows the Canonical Correspondence Analysis (CCA) (p-value = 0.001) and the richness and diversity measures calculated between species (as the average observed OTUs and Shannon diversity index of each sample), including all *Delphinus delphis* (n = 18), *Phocoena phocoena* (n = 10) and *Stenella coeruleoalba* (n = 10) samples (Figure 1 in the manuscript). Panel b and c show the same analysis performed in duplicate, after randomly normalizing the size of each group to 10 animals. The CCA plots were obtained after subsampling the OTU table to even sequencing depth and at the bacterial species level, with Hellinger transformation of the abundances. The colour frames in both CCA plots group the samples according to the constrained variable. The major contributions of the "host species" variable are shown as % in the first and second component of the CCA plot (CCA1 and CCA2, respectively). The significance of the alpha-diversity metrics was tested with Kruskal-Wallis chi-squared test followed by pairwise Wilcoxon test between groups (n.s, statistically not significant).

References:

Bik, E.M., Costello, E.K., Switzer, A.D., Callahan, B.J., Holmes, S.P., Wells, R.S., *et al*. (2016) Marine mammals harbor unique microbiotas shaped by and yet distinct from the sea. *Nat Commun* **7**: 10516;

Guindon, S., Dufayard, J-F., Lefort, V., Anisimova, M., Hordijk, W., Gascuel, O. (2010) New algorithms and methods to estimate maximum-likelihood phylogenies: assessing the performance of PhyML 3.0. *Syst Biol* **59**:307–321;

Katoh, K. and Standley, D.M. (2013) MAFFT Multiple Sequence Alignment Software Version 7: improvements in performance and usability. *Mol Biol Evol* **30**:772–780;

Trifinopoulos, J., Nguyen, L-T., von Haeseler, A., Minh, B. Q. (2016) W-IQ-TREE: a fast online phylogenetic tool for maximum likelihood analysis. *Nucleic Acids Res* **44**:W232-W235;
